# Knowledge-Based Sensors for Controlling A High-Concentration Photovoltaic Tracker

**DOI:** 10.3390/s20051315

**Published:** 2020-02-28

**Authors:** Joaquin Canada-Bago, Jose-Angel Fernandez-Prieto, Manuel-Angel Gadeo-Martos, Pedro Perez-Higueras

**Affiliations:** 1 Telematic Engineering System Research Group, CEATIC Center of Advanced Studies in Information and Communication Technologies, University of Jaén, Campus Las Lagunillas, C.P. 23071 Jaén, Spain; jan@ujaen.es (J.-A.F.-P.); gadeo@ujaen.es (M.-A.G.-M.); 2 CEAEMA Center for Advanced Studies in Energy and Environment, Electronic and Automation Department, University of Jaén, Campus Las Lagunillas, C.P. 23071 Jaén, Spain; pjperez@ujaen.es

**Keywords:** knowledge-based sensor, Internet of Things, high-concentration photovoltaic systems, sun tracker

## Abstract

To reduce the cost of generated electrical energy, high-concentration photovoltaic systems have been proposed to reduce the amount of semiconductor material needed by concentrating sunlight using lenses and mirrors. Due to the concentration of energy, the use of tracker or pointing systems is necessary in order to obtain the desired amount of electrical energy. However, a high degree of inaccuracy and imprecision is observed in the real installation of concentration photovoltaic systems. The main objective of this work is to design a knowledge-based controller for a high-concentration photovoltaic system (HCPV) tracker. The methodology proposed consists of using fuzzy rule-based systems (FRBS) and to implement the controller in a real system by means of Internet of Things (IoT) technologies. FRBS have demonstrated correct adaptation to problems having a high degree of inaccuracy and uncertainty, and IoT technology allows use of constrained resource devices, cloud computer architecture, and a platform to store and monitor the data obtained. As a result, two knowledge-based controllers are presented in this paper: the first based on a pointing device and the second based on the measure of the electrical current generated, which showed the best performance in the experiments carried out. New factors that increase imprecision and uncertainty in HCPV solar tracker installations are presented in the experiments carried out in the real installation.

## 1. Introduction

The European Commission has recently published the photovoltaic (PV) status report [1] in which PV market, electricity costs, and the economics of PV systems are analyzed. Within its conclusions, the following stand out: (a) the new installed capacity of solar PV power and the number and volume of PV markets are increasing; (b) a rapid decarbonization is necessary; (c) a rapid cost reduction exists in PV manufacturing; (d) different studies about subsidies for combustibles, fuels, and electricity have been presented; (e) solar energy will continue to grow at high rates; and (f) electricity from PV systems could be cheaper than residential consumer prices in a wide range of countries. 

To analyze the PV system profitability, it is convenient to take into account additional factors such as subsidies and forecasting of PV power generation. According to [1], while fossil fuel subsidies could indirectly increase noxious and greenhouse gases, renewable energies and energy efficient technologies subsidies may help to reduce emissions. A new scheme of subsidies based on the price of CO_2_ is presented in the literature [2]. A review of forecasting of PV generation is presented in the literature [3].

High-concentration photovoltaic systems (HCPVs) [4,5,6,7] concentrate the sunlight received between 300 and 2000 times onto photovoltaic cells by means of optical concentration devices. The main objective of these systems is to replace semiconductor materials (photovoltaic cells) with more economical optical materials (lenses and mirrors), reducing the cost of power plants and generated energy. 

Although HCPV is a young technology, it has already demonstrated a great capacity for growth in recent years. In this sense, the number of companies that develop HCPV systems has grown rapidly, and the installed power has gone from a few kWs in laboratories to several megawatts. 

According to [8] concentration photovoltaic (CPV) has potential for reducing the levelized cost of electricity. In this sense, if installations continue growing, CPV could reach a cost ranging between €0.045/kWh and €0.075/kWh. The system prices, including installation for CPV power plants, would then be between €700 and €1100/kWp. On the other hand, HCPV could be competitive in some locations in 2020 [9].

Due to the concentration of energy, tracker or pointing systems [10] are necessary in CPV and HCPV systems, which represents one of the differences with respect to conventional photovoltaic (PV) systems [11,12]. In these systems, power generation decreases dramatically with a sun pointing error greater than 0.5°, becoming practically zero if the error exceeds even a few degrees.

Frequently, a high degree of inaccuracy and uncertainty or imprecision are observed in HCPV tracker installations due to factors such as HCPV module manufacturing errors, module alignment errors, and the precision and accuracy of the tracker control system [13].

Fuzzy rule-based systems (FRBSs) [14] have demonstrated correct adaptation to problems having a high degree of inaccuracy and uncertainty. Based on fuzzy logic (FL) [15], these systems express knowledge by means of a set of linguistic rules grouped in a knowledge base (KB). FRBSs can be used in control systems, e.g., fuzzy logic controllers (FLCs), in which the control algorithm is expressed as a set of actuation linguistic rules.

Currently, there is a persistent trend to integrate knowledge-based systems (e.g., FRBSs) and FLCs into resource-constrained devices and into the paradigm of the Internet of Things (IoT) [16].

The IoT concept was introduced by Kevin Ashton in 1999, in which the physical world is connected to the Internet through ubiquitous sensors [17,18]. The IoT refers to the use of constrained resource devices, data acquisition, actuation, data communication with fog and cloud servers, data storage, and subsequent analysis. The range of applications of the IoT is very wide and includes environmental monitoring systems, fire detection, intelligent buildings, smart cities (traffic, lighting, parking location, garbage containers, etc.), intelligent agriculture, industrial control and monitoring, logistics, health monitoring, etc.

The main objective of this work is to design a knowledge-based controller for an HCPV tracker and to implement it in a real system by means of IoT technology (i.e., constrained resource devices, data communication, and a cloud computing server for data storage and analysis).

The remainder of this paper is organized as follows. The following section shows related work. Section 3 addresses the proposed controller and knowledge-based FRBS sensors. Section 4 presents the real HCPV tracker, the experiment that was carried out, and the results obtained. Finally, conclusions and future work are presented in Section 5.

## 2. Related Work and Background 

To achieve the objective, this work proposes to use FRBS due to the high degree of inaccuracy and uncertainty presented in real installations of PV trackers and IoT technologies to integrate the tracker controller into a resource-constrained device and monitor the data obtained. The following sections show the related work about PV trackers, and an introduction to FRBS and IoT technologies. 

### 2.1. PV Trackers

To point to the sun, PV installations use solar tracking systems or trackers that are composed of a metal structure that may be moved (whether on a dual or single axis) using motors, PV modules, position sensors, and a control system.

A review of different conventional PV tracker systems was presented in the literature [19], in which they are classified as active or passive control systems. The most common are active control systems, which may be differentiated into five types:

(a) Open–closed loop driver systems. The main difference of this type of controller is based on the feedback loop, which broadly uses closed loop systems that use the output variables as inputs to the control system.
(b) Sensor driver systems. These controllers are based on different devices (electronic components, sensors, or probes), such as electro-optical sensors, light-dependent resistors (LDRs) [20], light intensity sensors [21], and pointing devices.
(c) Microprocessor driver systems. The information processing capacity of the microprocessor allows the controller to execute algorithms such as calculating the sun position. The ephemeris algorithm presented in the literature [22] precisely calculates the azimuth and elevation angles of sun position using time, date, and tracker global position.
(d) Intelligent driver systems. These controllers are based on artificial intelligence technologies (using personal computers), such as neural networks and FL [23,24,25,26,27].
(e) Combination of sensors and microprocessors. The last type of controller is based on a hybrid system of sensors and microprocessors. Reference [11] presents a CPV hybrid controller with different strategies and an auto calibration system.

The main objective of controllers is to generate the maximum energy stabilizing the PV system in the maximum power point (MPP) by means of the maximum power point tracking (MPPT) technique [28,29] that is widely used in PV systems. A MPPT tracking based on learning is presented in [30]. To verify the proper operation of PV systems it is necessary to monitor the evolution of the significant magnitudes involved in the system [31,32,33].

Despite the wide interest in PV tracker controllers, little attention has been paid to tracker controllers for HCPV systems.

When HCPV systems are used, the sun pointing error has a maximum admitted value. The electrical current generation surface presents a maximum if azimuth and elevation errors are zero. If these errors are greater, the electrical current generated by the module decreases dramatically [34]. For example, the HCPV modules used in the experiments in this work require azimuth and elevation errors lower than ± 0.6°.

Although the algorithms and pointing devices are able to calculate the solar position with sufficient accuracy for HCPV systems, there is a high degree of inaccuracy and uncertainty or imprecision in HCPV tracker installations due to multiple factors. According to [13], there are three factors that cause system mismatches and power losses: manufacture error in HCPV modules, alignment error in the installation of the modules, and imprecision and inaccuracy in the tracker control system.

Due to these errors, the maximum power generation does not coincide with the zero error pointing of the tracker [35] in the installation of HCPV modules; therefore, controllers based on pointing algorithms (e.g., ephemeris) and pointing devices that minimize pointing error may present unacceptable errors in HCPV systems. However, the precision used in CPV installations is lower than that required in HCPV systems.

As a consequence, current trackers are not properly adapted to HCPV systems. These systems require more complex controls in order to obtain the maximum energy. Due to the optical concentration, the complexity of these systems, and the high degree of inaccuracy and uncertainty observed in the installation of these systems, a greater degree of precision and complexity in the control of tracker systems is necessary.

### 2.2. Fuzzy Rule-Based Systems

**Figure 1:**
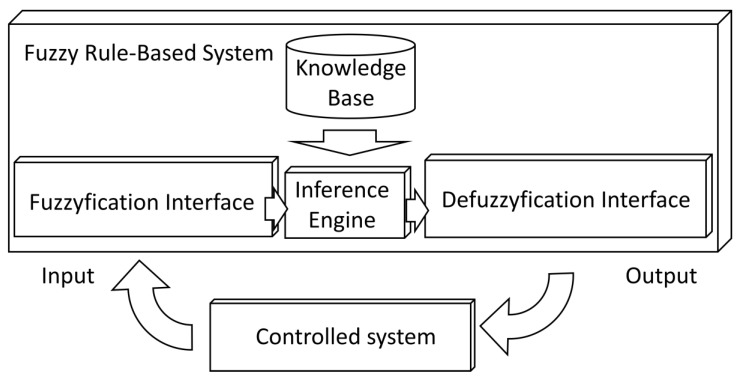
Fuzzy rule-based system.

A technology that has been demonstrated to adapt correctly to environments with inaccuracy and uncertainty is the FRBS [8], which uses FL and expresses knowledge through IF-THEN-type linguistic rules. These systems (Figure 1) are composed of a fuzzification interface, a KB, an inference engine, and defuzzification interface. The fuzzification interface adapts the actual input values to the fuzzy system. The KB contains the definition of input and output variables, the fuzzy sets defined in the variables, and a set of IF-THEN-type linguistic rules that corelate these variables. The inference engine is responsible for inferring the fuzzy output of the system from the input variables and the KB. Finally, the defuzzification interface adapts the value of the fuzzy output to a real output value.

Two approaches have been proposed within FRBSs: those of Mamdani [36,37] and Takagi– Sugeno–Kang (TSK) [38]. The main difference between the two approaches lies in the consequent knowledge rules. In the Mandani approach, the consequent is expressed as a linguistic variable:

IF X_1_ is A_1_ and . . . and X_n_ is A_n_ THEN Y is B,

where Xi represents input variables, A_i_ is fuzzy sets associated with input variables, Y is the output variable, and B is a fuzzy set associated with the output variable.

In the TSK approach, the consequent is an analytical function of the input variables:

IF X_1_ is A_1_ and . . . and X_n_ is A_n_ THEN Y = f (X_1_, . . . , X_n_).

where X_i_ represents input variables, A_i_ is fuzzy sets associated with input variables, Y is the output variable, and f (X_1_, . . . , X_n_) is the output function, in most cases, a linear function.

Controllers that use FRBS systems incorporating control knowledge are called FLCs (Figure 1).

In the literature [14], several FRBS applications are presented, such as classification systems, modeling systems, control systems, and robotics. A model of an HCPV module is presented using an FRBS system [39].

Currently, there is a trend to integrate knowledge-based systems into resource-constrained devices. Reference [40] presents a collaborative FRBS system for integration into wireless sensor networks (WSNs). In the literature [41], an optimization for smart spaces is proposed. Mariscal-Ramirez et al. [42] designed a sensor to monitor noise pollution adapted to resource-constrained devices.

### 2.3. Internet of Things

One of the objectives of this work is the use of IoT technologies to integrate the controller of an HCPV tracker into a resource-constrained device and monitor the data obtained using an IoT cloud platform.

Basically, IoT technologies [17,43] consist of constrained resource devices, data networks, communication protocols, and cloud platforms as follows:

(a) Although IoT devices have constrained resources, they have information processing capacity, sensor capacity of the environment, local information process, action on the environment, and the ability to communicate data with servers on the Internet. A first classification divides them into devices with an operating system (e.g., Raspberry) or without one (e.g., WaspMote, Arduino). These devices are used to obtain environmental data (temperature, pressure, etc.), detect alarms or extreme conditions (fire, gas leaks, etc.), or perform system control.
(b) Although there are data networks commonly used in IoT (IEEE 802.15.4, Long Rang (LORA), etc.), it is also possible to use conventional networks such as local area networks (LAN) (Ethernet, Wi-Fi, etc.) or mobile networks (4G).
(c) Specific IoT application protocols such as Message Queue Telemetry Transport (MQTT) or Constrained Application Protocol (CoAP) have been designed, although the use of other protocols such as HTTP is feasible.
(d) The data generated by IoT devices are sent to platforms for storage, visualization, analysis, and processing. Depending on the location of the servers, the platforms can be divided into cloud computing [44] and fog computing [45,46]. While cloud systems locate servers anywhere on the Internet, fog computing servers are closed to devices, usually on the same local network.

The use of IoT in smart spaces [18] and smart devices is widely referenced in European Commission documents [47,48] concerning the Internet of Things and the Internet of the Future. These documents present devices called smart things in which several algorithms can be executed for intelligent decisions based on real-time measurements of the sensors.

## 3. HCPV Tracker Knowledge-Based Controller

The main objective of this work is to design a knowledge-based controller for an HCPV tracker. Due to the uncertainty and inaccuracy of the positioning or tracker systems, this work proposes the use of FRBS systems because these knowledge-based systems have demonstrated their effectiveness in these conditions.

In addition, the proposed system will be implemented in a real system using IoT technology with constrained resource devices, data communication, and a platform for storing and analyzing the data obtained. Therefore, all the algorithms used in the control system will be designed to be executed in a low-cost microcontroller with low information processing capacity.

Therefore, the novelty of the proposed controller lies in (a) the design of a knowledge-based controller by means of FRBS, (b) easy to understand control knowledge, and (c) a design to be executed on a resource-constrained device. 

The following sections show the structure of the proposed controller and two knowledge-based FRBS sensors: the first uses a positioning device, and the second is based on the electrical current generated by the photovoltaic concentration modules.

### 3.1. Controller Structure

**Figure 2:**
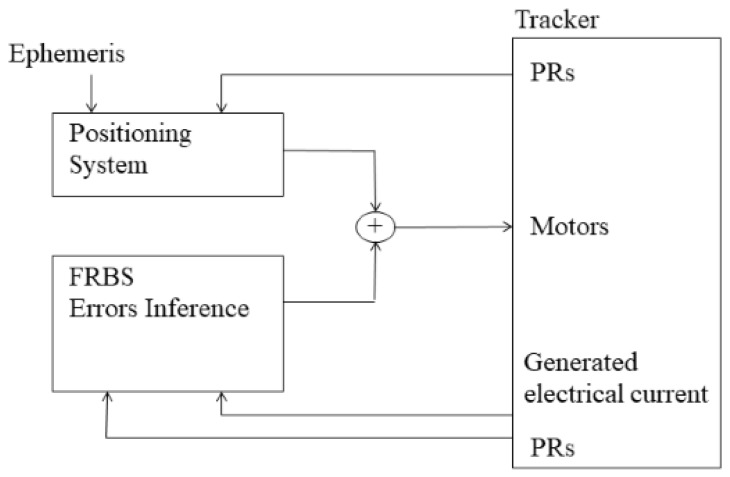
Controller structure.

Figure 2 shows the basic structure of the control system, which is composed of a pointing system, an error inference system, and a solar tracking system. In this way, the positioning of the tracker will be calculated as the sum of the positioning algorithm and the error inferred by the FRBS systems.

The calculation of the solar position can be performed by different algorithms (such as ephemeris) or by a solar position sensor. If an ephemeris algorithm is used, it calculates the position of the sun using the date, time, and global position of the solar tracker. After that step, the controller compares the position of the sun with the position to which the tracker is pointing and calculates the azimuth and elevation angles that the solar tracker has to perform in the next movement.

To calculate the pointing error, a knowledge-based FRBS is used. To execute the FRBS in a constrained resource device, we introduced several modifications to the classical structure of Mandani FRBS to minimize computational burden: the device executes a small but complete FRBS; only triangular fuzzy sets are available; fuzzification and defuzzification interfaces only admit linear conversions; a First Infer Then Aggregate (FITA) inference approach is used; the inference engine operates with numerical values instead of linguistic labels; and the number of fuzzy sets defined in each variable and rules in the KB is small.

This work presents two knowledge-based FRBSs to infer the pointing error. The first is a smart sensor that is composed of a pointing device and an FRBS that infers the error. The second one is a different smart sensor composed of a probe that obtains the electric current generated by the HCPV module and another FRBS to infer the error.

### 3.2. FRBS Sensor Based on a Pointing Device

This smart sensor is composed of a hardware pointing device that allows measuring luminosity by means of four photoresistors (PRs) and an FRBS that infers the pointing error using a specific KB.

The pointing device is composed of an optical light/shadow device, a set of four PRs, and a signal adaptation stage. In this way, the luminosity values measured by the PRs are the inputs to the FRBS that infer the error. If the sensor is pointed correctly to the sun, each of the PRs has the same solar radiation. In the case of a pointing error, the optical device increases the radiation difference that some PRs receive compared to others to detect small pointing errors.

**Figure 3:**
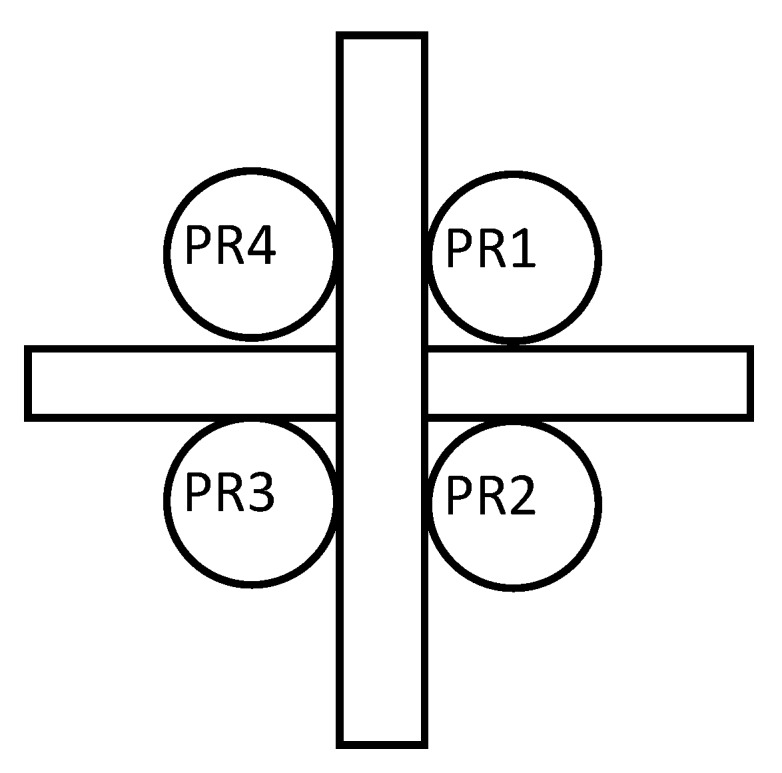
Pointing device. Top view.

**Figure 4:**
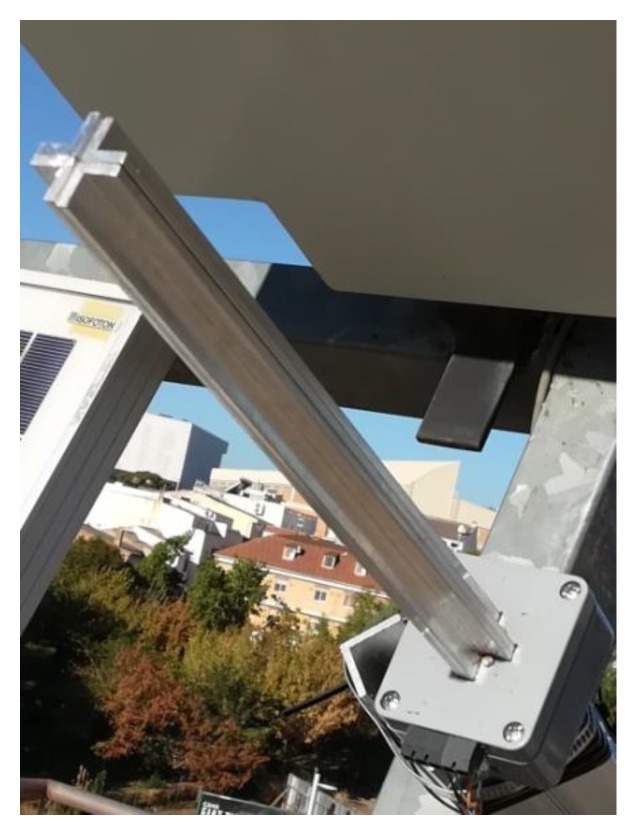
Pointing device.

Figure 3 and Figure 4 show the pointing device used by the sensor. The difference between the luminosity measured by PR1 and PR2 allows the sensor to infer the elevation error. Similarly, it can be estimated using PR4 and PR3, which allows the sensor to infer the error with two different systems. On the other hand, the difference in luminosity measured by PR4 and PR1 (as well as between PR3 and PR2) allows the sensor to infer the azimuth error.

This work proposes to use two KBs, one to infer the elevation error and another to infer the azimuth error. Since KBs will be executed in a constrained resource system, a small number of fuzzy sets defined in variables and action rules will be used to infer the error as quickly as possible.

**Figure 5:**
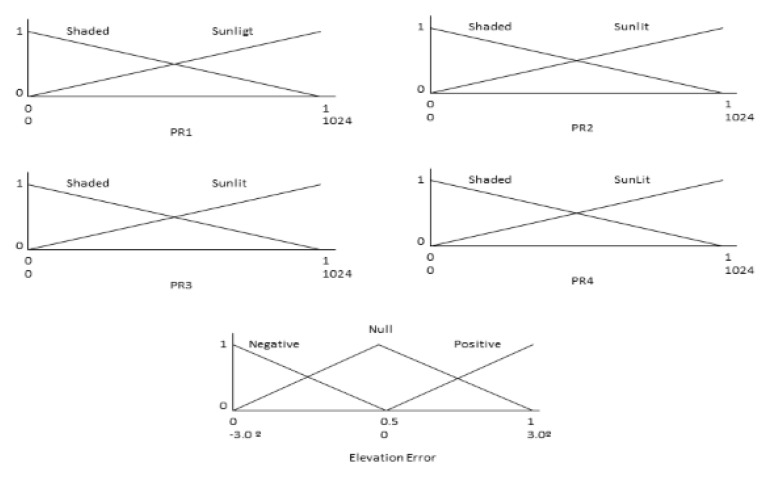
Fuzzy sets defined in input (PRs) and output variables (elevation error).

Elevation error KB consists of two input variables (luminosity measured in two PRs), one output variable (elevation error), and a set of action rules. Figure 5 shows the fuzzy sets defined for all input variables (PRs) and the output variable (elevation error).

Table 1 shows the KB elevation error action rules for the sensor using PR1 and PR2.

Table 2 shows the KB elevation error action rules for the sensor using PR4 and PR3.

The azimuth error KB uses the same definition of input and output variables as the elevation error KB (Figure 5). However, the set of action rules is different. Table 3 shows the KB azimuth error action rules for the sensor using PR4 and PR1.

Table 4 shows the KB elevation error action rules for the sensor using PR3 and PR2.

The fuzzification interface linearly converts the range [0, 1024] measured by the PRs to the normalized range [0, 1] and the output range [0, 1] to [−3°, 3°].

### 3.3. FRBS Sensor Based on the Electrical Current Generated

This sensor is composed of a hardware probe that measures the electrical current generated by HCPV modules and an FRBS, which infers the elevation and azimuth errors.

The controller uses the usual sun tracking movements to measure the electrical current generation before and after each movement. After each movement, these two measurements are used to infer the elevation and azimuth errors. The inferred errors are taken into account in the next tracker movement to correct the errors and follow the maximum electrical current generated. Therefore, no extraordinary movements are made.

**Figure 6:**
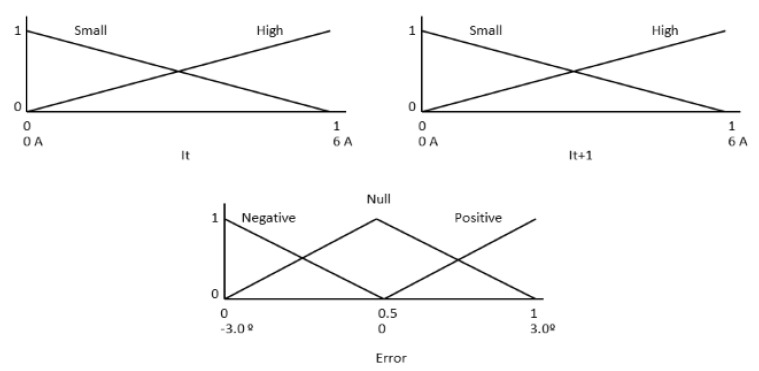
Fuzzy sets defined in input (It and I_t + 1_) and output variables (error).

The KB of this FRBS system is used to infer both the elevation and azimuth errors and is composed of two input variables (electrical current before and after the movement), an output variable (error committed), and a set of action rules. Figure 6 shows the fuzzy sets defined in input variables (I_t_ and I_t + 1_) and output variable (Error) (with an HCPV module with Imax 6A and a maximum error of ± 3°).

Table 5 shows the KB error action rules used by the sensor.

The fuzzification interface linearly converts the range [0, 6] measured by the electrical current probe to the normalized range [0, 1] and the output range [0, 1] to [−3°, 3°].

## 4. Experimental Results

To evaluate the controller and FRBS sensors proposed in the previous section, a real two-axis tracker with HCPV modules controlled by a low-cost microcontroller was designed and implemented. In addition, elevation and azimuth errors of tracker pointing with respect to the sun are measured by a precision instrument. On the other hand, data obtained (most significant tracker variables and elevation and azimuth errors) were sent to an IoT platform in order to analyze their evolution and compare the results of the different FRBS systems proposed.

This section describes the HCPV tracker used in the experiments carried out and the results obtained with the following controllers based on (a) an ephemeris algorithm only; (b) an FRBS sensor based on a pointing device; and (c) an FRBS sensor based on the electrical current generated.

### 4.1. HCPV Tracker

**Figure 7:**
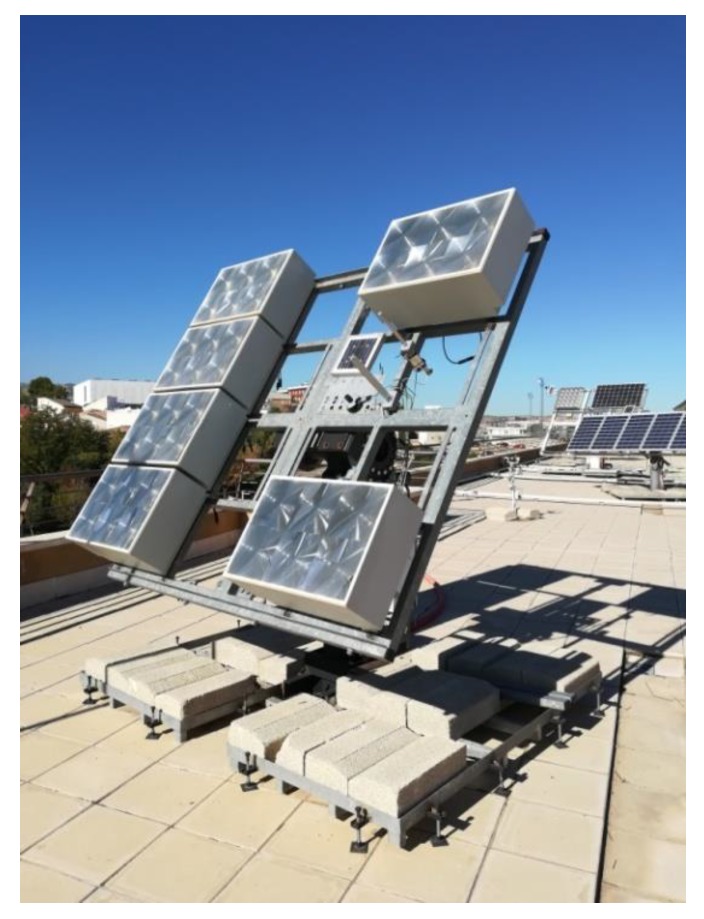
The designed high-concentration photovoltaic system (HCPV) tracker.

**Figure 8:**
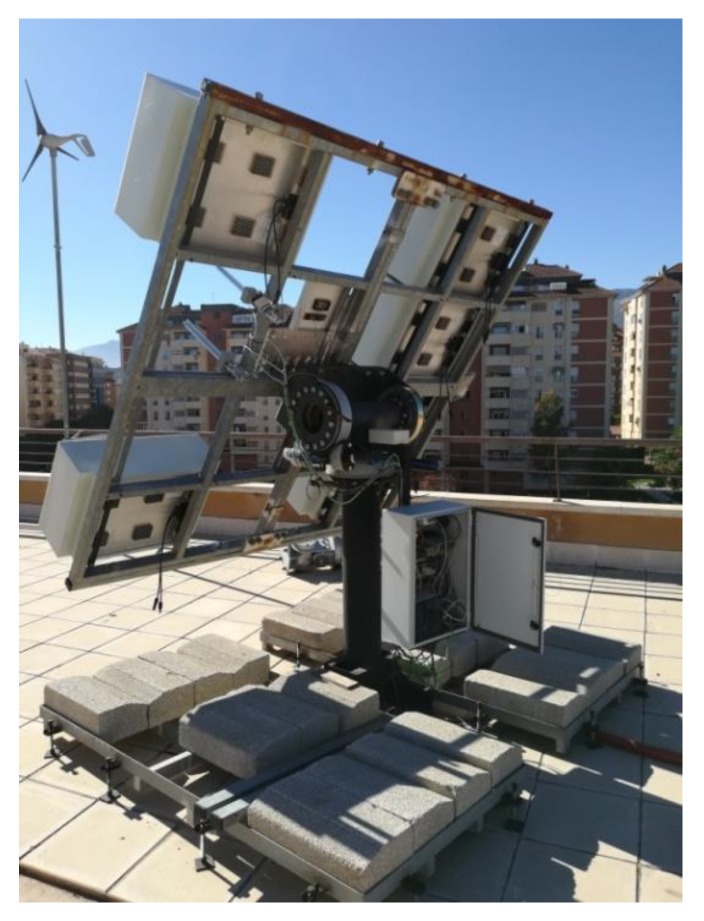
The designed high-concentration photovoltaic system (HCPV) tracker.

The two-axis solar tracker (Figure 7 and Figure 8) is composed of a metal structure with the possibility of movement in elevation and azimuth by means of gearboxes, various HCVPV modules, a calibrated solar cell, DC azimuth and elevation motors, a measurement system of the angular movement of each motor (encoders), a pointing error sensor, electrical current-generated sensors, and the control system.

**Figure 9:**
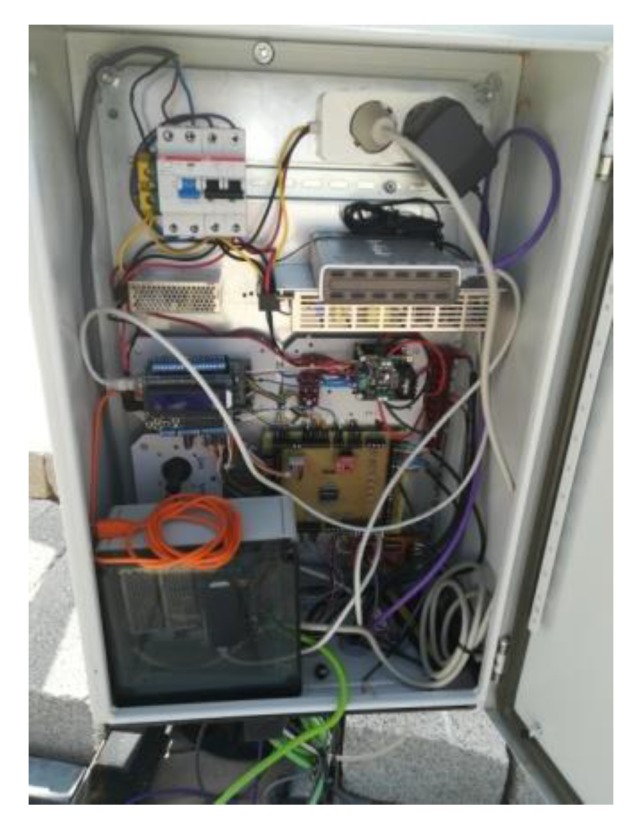
Controller.

The control system (Figure 9) is based on a low-cost 32-bit microcontroller and several signal adaptation interfaces to the following inputs and outputs:

- Nine analog inputs: four PRs, temperature, solar radiation, direction and speed wind sensors, and electrical current generated.
- Ten digital inputs: two encoders, four inputs for a joystick, and four limit switches.
- Two digital outputs: azimuth and elevation motors.

The controller calculates the elevation and azimuth angles to be performed at each moment using the state of the system (date, time, position of the sun, position of the tracker, etc.). On the other hand, the error inferred by the FRBS system is added to the calculated angles. The angular movements of elevation or azimuth of the tracker are carried out by means of an algorithm that calculates the activation and braking time of the motor as well as a maximum safety time. The movement made at each angle is verified by means of the encoders.

**Figure 10:**
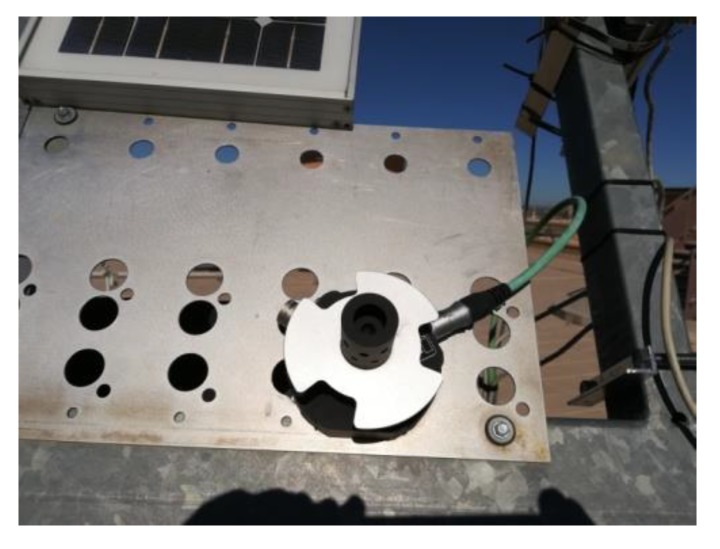
Tracking accuracy sensor.

To measure the real elevation and azimuth error of the tracker in the sun, a Black Photon Tracking Accuracy Sensor measuring instrument (Figure 10) is available. The instrument is able to measure elevation and azimuth errors in the range ± 1.2° with a resolution of 0.0005°. The data obtained from the instrument allow us to check the correct tracker pointing and are not used in tracker control.

Data generated by the system (sensor measurements, solar radiation, temperature, solar position, tracker position, electrical current generated, etc.) are sent to an Internet IoT cloud platform that stores the data and allows users to monitor the temporal evolution of all variables using a web browser.

The main characteristics of HCPV modules used in the tracker are the following (at 1000 W/m2, 25°C, AM1.5D): short-circuit current 6.35A, open-circuit voltage 18.45 V, DC power 95 W, and needed pointing error < ± 0.6°. 

**Figure 11:**
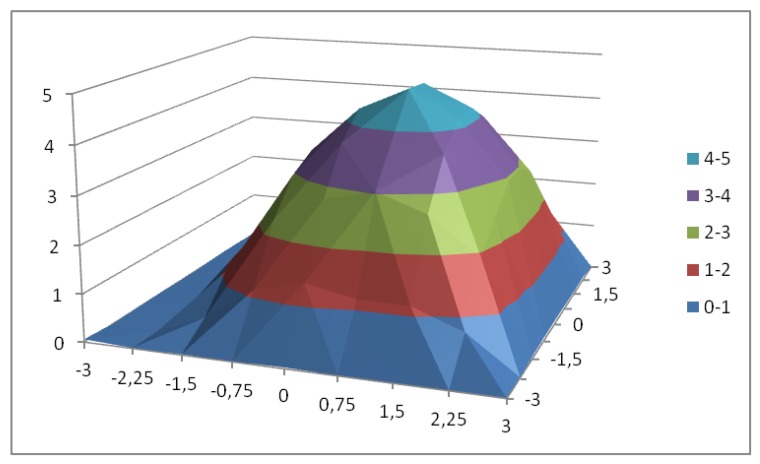
Electrical current generated in an elevation and azimuth sector of ±3°.

To characterize the HCPV module, a complete exploration was carried out by measuring the short-circuit electrical current generated by varying its position with respect to the sun in an angular sector of ± 3° in elevation and azimuth. Figure 11 shows the obtained surface where it is observed that the maximum electrical current generation is not at the 0° elevation and azimuth point. The maximum current (5.54 A) is at an elevation error of +0.2° and an azimuth error of −0.8°. On the other hand, the surface shows that the current generated falls drastically with a small variation of the elevation and azimuth angles.

### 4.2. Controller based on an Ephemeris Algorithm

The first part of the experiments carried out has the objective of measuring the effectiveness of an ephemeris algorithm applied to a real tracker in which different imprecision factors can exist according to the reasons stated in Section 2.

**Figure 12:**
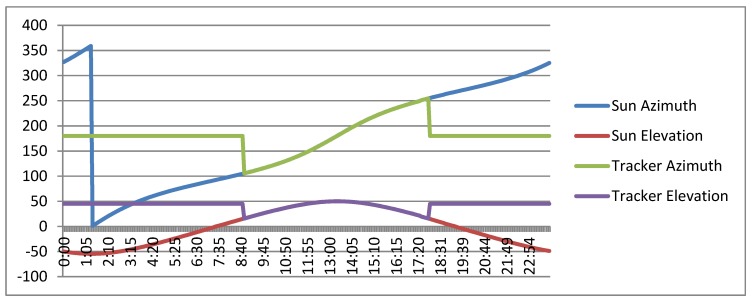
Sun position and tracker pointing (with both elevation and azimuth angles).

Figure 12 shows a simulation in which the position of the sun (angles of azimuth and elevation) is calculated by the ephemeris algorithm as well as the tracker pointing during a day without taking into account the elevation and azimuth errors. In the graph, it can be observed how the tracker would be pointing at the sun practically without error during the period of time in which the sun elevation was greater than 15°. The rest of the day, the tracker would be in a resting position (45° elevation, 180° azimuth).

**Figure 13:**
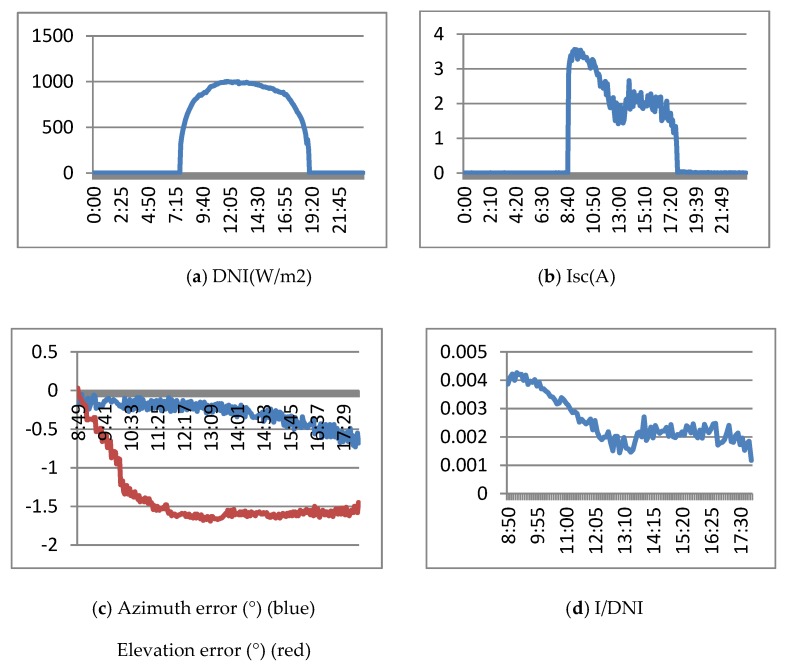
Results of the ephemeris controller. March 15, 2019.

Figure 13 shows the results obtained when the real tracker is controlled with the ephemeris algorithm. Figure 13a shows the DNI (direct normal irradiance) of March 15, 2019. It is a sunny day with a maximum of 1000 w/m2. Figure 13b shows the Isc (short circuit current) obtained by the HCPV module. Figure 13c shows the evolution of elevation and azimuth errors (measured by the precision instrument) with respect to the sun. Finally, Figure 13d presents the Isc/DNI ratio, which represents a normalization that allows comparison of the currents generated on different days. Figure 13c,d shows the results from approximately 9:00 a.m. at 6:00 p.m. which corresponds to an elevation of the sun greater than 15°.

Figure 13c shows how at the beginning of the experiment (9 am), it starts with an error of 0° in elevation and azimuth errors and the way in which both errors are increasing and exceeding the values recommended by the manufacturer (elevation error). Due to these errors, the generated current decreases in the first part of the day, although it is stabilized at the end. As a result of the elevation and azimuth pointing errors, the concentration generator does not operate at maximum, and the current generated is much lower than expected.

### 4.3. Controller Based on an FRBS Sensor with a Pointing Device

To improve the performance of the photovoltaic generator and correct the evolution of elevation and azimuth errors, this work proposes the use of an economic sensor based on an FRBS system and a pointing device that infers the elevation and azimuth errors.

**Figure 14:**
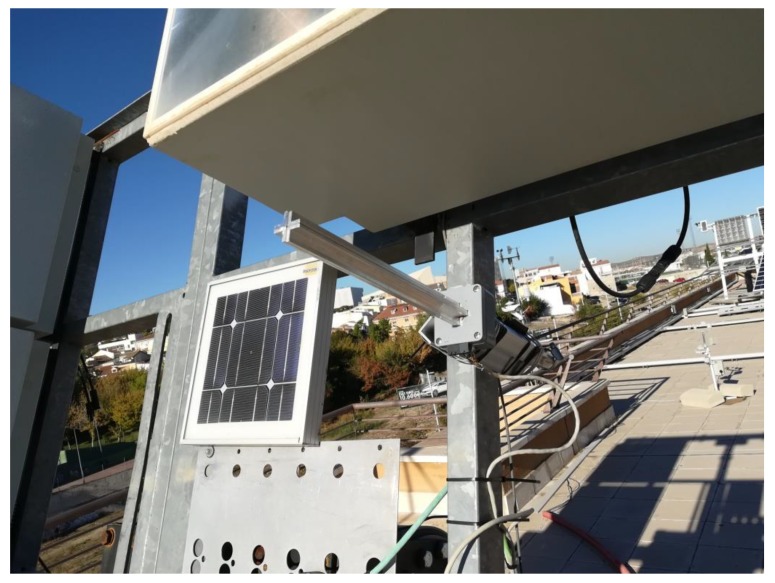
Pointing device.

This controller is composed of an optical light/shadow pointing device, a set of four PRs, a signal adaptation stage and an FRBS system. In the case of a pointing error, the pointing device increases the difference in radiation received by some PRs compared to others to detect small pointing errors. The pointing sensor is based on the device shown in Figure 14, and the FRBS system is shown in Figure 5 and Table 1, Table 2, Table 3 and Table 4.

Each PR of the pointing sensor is able to measure the luminosity that they are receiving by means of modifying their electrical resistance. Through an adaptation stage, the microcontroller can measure a proportional voltage (by means of a 12-bit analog input) in such a way that a value of 1,023 is obtained in the maximum luminosity and 0 in the dark.

**Figure 15:**
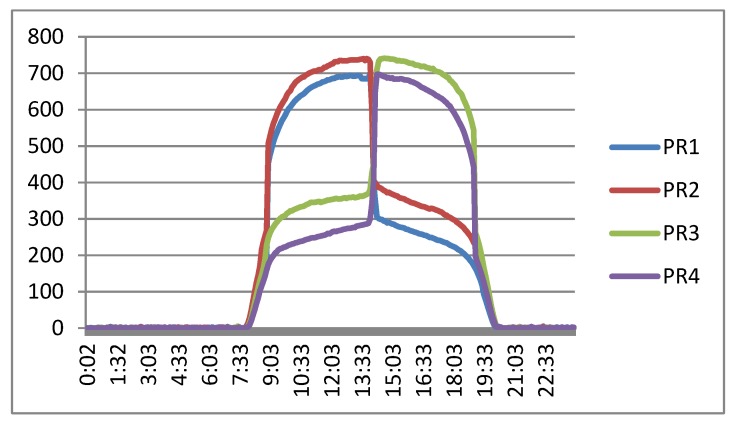
Evolution of the luminosity value obtained in the four PRs.

Figure 15 shows the evolution of the luminosity value obtained in the four PRs. The data in the figure have been obtained with the tracker stopped while pointing to the sun with an approximate error of 0° in elevation and azimuth at a certain time (2:00 p.m). In this way, the evolution of the luminosity measured can be observed during a full sunny day.

**Figure 16:**
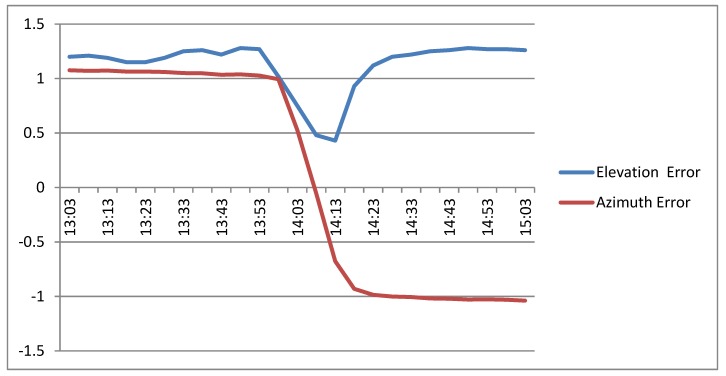
Elevation and azimuth errors inferred by the FRBS.

Figure 16 shows the error inferred by the FRBS system between 13:00 and 15:00. In the initial and final parts, the sensor infers a constant error: a positive degree of error in elevation and azimuth in the initial part and a positive degree in elevation and a negative degree in azimuth. In the central part, the figure shows the inferred error when the device is pointing to the sun, inferring that the sensor has not been correctly oriented (minimum inferred error +0.5° elevation, 0° azimuth).

**Figure 17:**
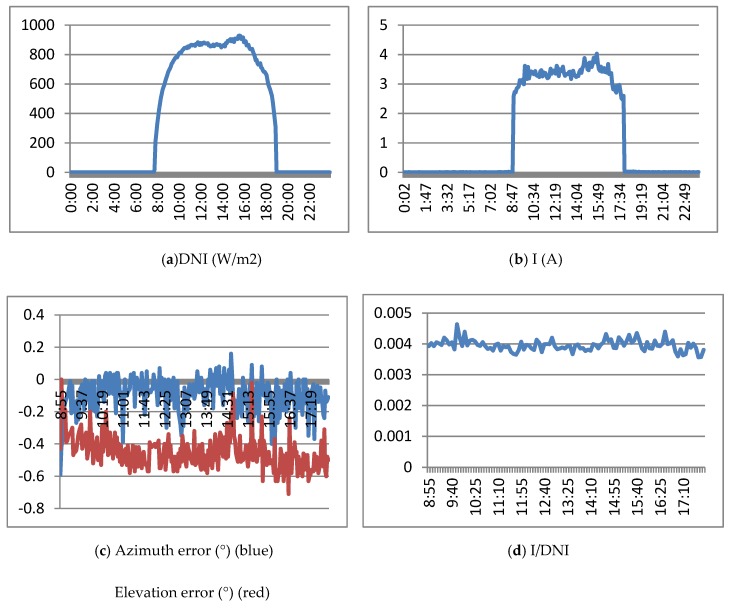
Results of the controller based on an FRBS sensor with the pointing device. March 12, 2019.

Figure 17 shows the results obtained when the tracker is controlled by the ephemeris algorithm modified with the FRBS sensor with the pointing device. Figure 17a shows the DNI of March 12, 2019. It is a sunny day with a maximum of 850 w/m2. Figure 17b shows the Isc current obtained by the HCPV module. Figure 17c shows the evolution of elevation and azimuth errors (measured by the precision instrument) with respect to the sun. Finally, Figure 17d presents the Isc / DNI ratio. Figure 17c,d shows the results from approximately 9:00 a.m. at 6:00 p.m. which corresponds to a sun elevation greater than 15°.

Figure 17 shows the following:

(a) Although the maximum electrical current generation (obtained in the characterization of the HCPV module) is not achieved, the generated electrical current is greater when using the controller based on an FRBS sensor with the pointing device.
(b) The elevation and azimuth errors of this controller are quite minor compared to the errors observed in the controller based on an ephemeris algorithm. The average values of the errors measured by the instrument are −0.10° for the azimuth and −0.45° for the elevation, closer to those of the characterization.
(c) The Isc/DNI ratio shows a performance improvement when using this control system.

### 4.4. Controller based on an FRBS Sensor based on the Electrical Current Generated

Although the inference of the pointing error improves the electrical current generated by the HCPV module, it does not obtain the maximum current generated.

In this section, an FRBS based on the electrical current generated is proposed in order to modify the position of the tracker obtained by the ephemeris algorithm. The controller proposed is based on the calculation of the pointing error by means of the FRBS system described in Section 3.3. In this controller, each time the tracker moves in elevation or azimuth (by means of ephemeris), the pointing error is inferred. The error is corrected in the next movement so that no extraordinary movements are made exclusively to correct the error.

**Figure 18:**
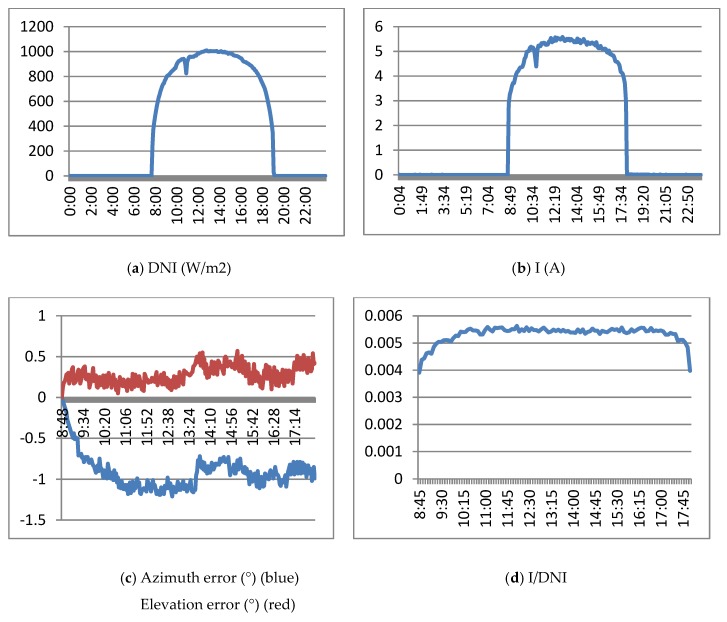
Results of the controller based on the FRBS sensor based on the electrical current generated. 14 March 2019.

Figure 18a shows the DNI of March 14, 2019. It was a sunny day with a maximum of 1000 w/m2. Figure 18b shows the Isc electrical current obtained by the HCPV module. Figure 18c shows the evolution of elevation and azimuth errors (measured by the precision instrument) with respect to the sun. Finally, Figure 18d presents the Isc / DNI ratio, which represents a normalization that allows comparison of the currents generated on different days. Figure 18c,d shows the results from approximately 9:00 a.m. to 6:00 p.m., which correspond to an elevation of the sun greater than 15°.

Figure 18 shows the following:

(a) The generated current is greater than that obtained with previous controllers, with a value very similar to the value obtained in the characterization of the module. On the other hand, there is less ripple in the current in this case.
(b) Although the electrical current is stabilized and the Isc/DNI ratio is practically flat, it should be noted that the error does not remain constant throughout the day. The control method dynamically modifies the error and tracker position in order to obtain the highest current.
(c) The average values of the errors measured by the instrument (average azimuth −0.91°; average elevation 0.27°) are very similar to those obtained in the characterization of the module (azimuth error −0.8°; elevation error +0.2°).
(d) The Isc/DNI ratio shows a performance improvement over the other control methods.

The main benefits of the proposed controller are as follows:

- The controller showed the best performance, near the maximum current measured in the module characterization;
- A knowledge-based controller with correct adaptation to inaccuracy and uncertainty and easy to understand knowledge;
- The controller can be executed on a resource-constrained cheap device, using IoT technology. A summary of the controller cost is as follows: 32 bits microcontroller—€20, wind sensors—€20, calibrated solar cell—€7, electrical current sensor—€4, miscellaneous material (photoresistors, temperature probe, etc.)—€2;
- It dynamically modifies the tracker position to obtain near the maximum of generated electrical energy with minimal oscillation.

## 5. Conclusions

IoT technologies were able to execute HCPV controllers and monitor the evolution of variables in a satisfactory way. The constrained resource microcontroller executed the knowledge-based controllers with response times shorter than needed in the application. Although there was some timely loss of data due to the unavailability of communication on the Internet, the cloud computing architecture used in the project was more than sufficient. Data obtained in the system was correctly stored in the platform and monitored by users

Additional factors were presented to those provided in the literature [13], which increase imprecision and uncertainty in HCPV solar tracker installations. The factors presented in the project are the following:

(a) Inaccuracy in the manufacturing process of HCPV modules in relation to the alignment of the different components;
(b) Precision errors in the manufacture of the tracker structure;
(c) Precision errors in the installation of tracker structure (e.g., wrong leveling);
(d) Precision errors in the installation of HCPC modules in the tracker;
(e) Minimum movement of the electrical engines, which may be greater than necessary in some instances;
(f) Inaccurate movement of the electrical engines;
(g) Low resolution of encoders that measure the angle performed by electrical engines;
(h) Other factors such as wind.

The characterization of the HCPV module installed in the tracker verifies that the maximum energy may not be in the zero pointing error of the tracker due to the imprecision and uncertainty factors. In addition, to generate the maximum electrical current, it must be taken into account that a pointing error less than 0.6° is necessary. 

The controller based exclusively on the ephemeris algorithm obtains very low performance due to the accumulation of azimuth and elevation errors. In this case, the tracker leveling error was important. When using this kind of controller, it would be necessary to calculate the error made and take it into account in the control algorithm.

The controller based on the FRBS sensor with a pointing device infers the azimuth and elevation error and increases the generated electrical current, improving the performance of the exclusively ephemeris-based controller. This controller requires that the maximum electrical current generation of the HCPV module installed in the tracker and the pointing device be perfectly calibrated (pointing to the same exact angle). In this case, a periodic calibration would be necessary.

The controller based on the FRBS sensor and an electrical current probe showed the best performance, obtaining values similar to those obtained in the module characterization. In this case, calibration is not necessary since the algorithm dynamically locates the maximum current generation.

Regarding future work, we propose the following actions: to use an IoT fog computing architecture in order to avoid punctual data loss; to characterize different HCPV modules in the real tracker; to compare other controllers; and to characterize HCPV systems composed of several modules in order to locate their maximum current generation.

## Figures and Tables

**Table 1:** Action rules for the elevation error (PR1 and PR2).

Rule		Antecedents		Consequent
R1	If	PR1 is shaded and PR2 is shaded	then	the elevation error is null
R2	If	PR1 is shaded and PR2 is sunlit	then	the elevation error is positive
R3	If	PR1 is sunlit and PR2 is shaded	then	the elevation error is negative
R4	If	PR1 is sunlit and PR2 is sunlit	then	the elevation error is null

**Table 2:** Action rules for the elevation error (PR4 and PR3).

Rule		Antecedents		Consequent
R1	If	PR4 is shaded and PR3 is shaded	then	the elevation error is null
R2	If	PR4 is shaded and PR3 is sunlit	then	the elevation error is positive
R3	If	PR4 is sunlit and PR3 is shaded	then	the elevation error is negative
R4	If	PR4 is sunlit and PR3 is sunlit	then	the elevation error is null

**Table 3:** Action rules for the azimuth error (PR4 and PR1).

Rule		Antecedents		Consequent
R1	If	PR4 is shaded and PR1 is shaded	then	the azimuth error is null
R2	If	PR4 is shaded and PR1 is sunlit	then	the azimuth error is positive
R3	If	PR4 is sunlit and PR1 is shaded	then	the azimuth error is negative
R4	If	PR4 is sunlit and PR1 is sunlit	then	the azimuth error is null

**Table 4:** Action rules for the azimuth error (PR3 and PR2).

Rule		Antecedents		Consequent
R1	If	PR3 is shaded and PR2 is shaded	then	the azimuth error is null
R2	If	PR3 is shaded and PR2 is sunlit	then	the azimuth error is positive
R3	If	PR3 is sunlit and PR2 is shaded	then	the azimuth error is negative
R4	If	PR3 is sunlit and PR2 is sunlit	then	the azimuth error is null

**Table 5:** Action rules in knowledge base (KB) error.

Rule		Antecedents		Consequent
R1	If	I_t_ is low and I_t+1_ is low	then	the error is null
R2	If	I_t_ is low and I_t+1_ is high	then	the error is negative
R3	If	It is high and I_t+1_ is low	then	the error is positive
R4	If	I_t_ is high and I_t+1_ is high	then	the error is null
